# Function-Related Asymmetry of the Interactions between Matrix Loops and Conserved Sequence Motifs in the Mitochondrial ADP/ATP Carrier

**DOI:** 10.3390/ijms231810877

**Published:** 2022-09-17

**Authors:** Qiuzi Yi, Shihao Yao, Boyuan Ma, Xiaohui Cang

**Affiliations:** 1 Division of Medical Genetics and Genomics, The Children’s Hospital, Zhejiang University School of Medicine, Hangzhou 310052, China; 2 Institute of Genetics, Department of Genetics, Zhejiang University School of Medicine, Hangzhou 310058, China; 3 Zhejiang Provincial Key Laboratory of Genetic and Developmental Disorder, Hangzhou 310058, China

**Keywords:** ADP/ATP carrier (AAC), mitochondrial carrier family (MCF), transporters, loops, MCF motif, molecular dynamics simulation

## Abstract

The ADP/ATP carrier (AAC) plays a central role in oxidative metabolism by exchanging ATP and ADP across the inner mitochondrial membrane. Previous experiments have shown the involvement of the matrix loops of AAC in its function, yet potential mechanisms remain largely elusive. One obstacle is the limited information on the structural dynamics of the matrix loops. In the current work, unbiased all-atom molecular dynamics (MD) simulations were carried out on c-state wild-type AAC and mutants. Our results reveal that: (1) two ends of a matrix loop are tethered through interactions between the residue of triplet 38 (Q38, D143 and Q240) located at the C-end of the odd-numbered helix and residues of the [YF]xG motif located before the N-end of the short matrix helix in the same domain; (2) the initial progression direction of a matrix loop is determined by interactions between the negatively charged residue of the [DE]G motif located at the C-end of the short matrix helix and the capping arginine (R30, R139 and R236) in the previous domain; (3) the two chemically similar residues D and E in the highly conserved [DE]G motif are actually quite different; (4) the N-end of the M3 loop is clamped by the [DE]G motif and the capping arginine of domain 2 from the two sides, which strengthens interactions between domain 2 and domain 3; and (5) a highly asymmetric stable core exists within domains 2 and 3 at the m-gate level. Moreover, our results help explain almost all extremely conserved residues within the matrix loops of the ADP/ATP carriers from a structural point of view. Taken together, the current work highlights asymmetry in the three matrix loops and implies a close relationship between asymmetry and ADP/ATP transport.

## 1. Introduction

**Figure 1:**
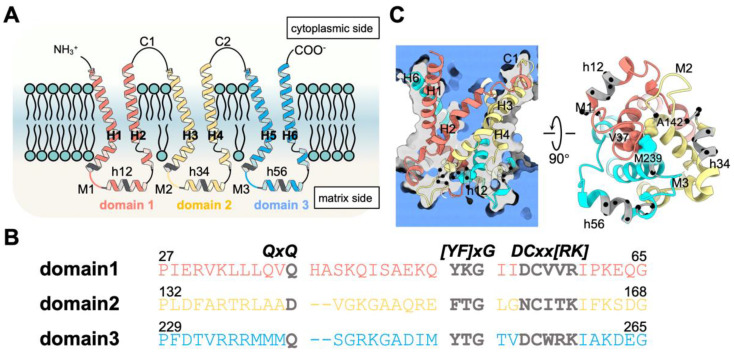
The matrix loops in AAC and conserved MCF motifs nearby. (**A**) Schematic diagram of the tripartite structure of AAC. Each domain is shown in a different color (domain 1—salmon; domain 2—yellow; domain 3—cyan). The [YF]xG and DCxx[RK] motifs and triplet 38 (Q38, D143 and Q240) within the Px[DE]xx[KR]xRxQxQ motif are highlighted in gray. (**B**) Sequence alignment of the three domains from triplet 27 (conserved kink prolines) to triplet 65 (the G of the [DE]G motif at the C-end of short matrix helices h12, h34 and h56), with residues numbered based on bovine AAC1. The motif residues mentioned above are highlighted in gray characters. (**C**) The tertiary structure of AAC in the c-state. The left panel represents a cross-section of AAC and solvent, with the interior of AAC shown in gray and the solvent shown in blue. Cα atoms of the motifs mentioned above are highlighted by small black balls, and residues in the hydrophobic plug (V37, A142 and M239) at the C-end of odd-numbered helices are shown as spheres.

The mitochondrial carrier family (MCF), also known as SLC25, represents the largest group of the solute carrier superfamily. To date, 53 mitochondrial carriers (MCs) have been identified in the human genome, and almost all of them are located in the inner mitochondrial membrane (IMM), with some peculiar members localized to the outer mitochondrial membrane (MTCH1, MTCH2 and SLC25A46) [1,2,3] or the peroxisomal membrane (PM34) [4,5]. These carriers transport a variety of metabolites, including nucleotides, amino acids, carboxylic acids, cofactors and inorganic anions, across the IMM and are crucial for the normal function of mitochondria [6]. Defects in the carriers are linked to diseases such as severe obesity, type II diabetes, neonatal myoclonic epilepsy, Amish-type microcephaly and type II citrullinemia [6]. The MCs are unique among transporters for their tripartite folding topology [7,8]. Each of the three homologous repeat domains is 100 aa long, forming two transmembrane helices connected by one matrix loop and one short matrix helix, with two adjacent domains connected through cytoplasmic loops (Figure 1A). These homologous repeat domains are highly conserved in their primary sequences, indicating that MCs share similar 3D structures and possibly similar transport mechanisms.

The mitochondrial ADP/ATP carrier (AAC) is a paradigm of the big MCF family [9]. It exchanges ADP and ATP in a strict equal molar ratio by alternating between the cytosol-open (c-) and matrix-open (m-) conformations [10]. AAC in the c-state specifically recognizes and imports ADP [11], while AAC in the m-state specifically recognizes and exports ATP. AAC is also the only MCF member whose high-resolution crystal structures have been solved [12,13,14]. In the c-state structure of AAC, six transmembrane helices surround a cone-shaped transport cavity opening wide toward the intermembrane space, and the whole molecule exhibits three-fold rotational pseudosymmetry [12,13,14,15] (Figure 1C). In the m-state structure, which opens toward the matrix side, the cytoplasmic side is three-fold pseudosymmetric, but the matrix side is asymmetric, with domain 1 displaced from the other two domains [15].

Mitochondrial carriers share conserved sequence motifs such as the [YF][DE]xx[RK] motif, the GxxxG motif and the πxxxπ motif (here, π represents a small residue) near the cytoplasmic side in each domain [16] and a more conserved MCF motif near the matrix side: Px[DE]xx[KR]xRxQxQ-(matrix loop-matrix helix)-[DE]Gxxxx[YWF][KR]G [8,17,18,19,20,21], with the short matrix helix hosting the sequence motif [YF]xGxxDCxx[RK] [8,14,22] (Figure 1B). The [YF][DE]xx[RK] motifs are located on the even-numbered helices and host residues forming the cytosolic gate in the m-state [7,8,15]. The GxxxG motifs on the odd-numbered helices and the πxxxπ motifs on both odd-numbered and even-numbered helices are proposed to be involved in the helix–helix association and conformational transition [8,14,22,23]. The Px[DE]xx[KR]xRxQxQ motifs are located on the matrix side of the odd-numbered helices, with P (P27, P132, P229) inducing sharp kinks, [DE]xx[KR] forming the matrix gate in the c-state, the capping arginine R (R30, R139 and R264) stabilizing the C-ends of the odd-numbered helices and the first Q forming the glutamine brace within the matrix gate [9,12,14,22]. The [YF]xG motif and [DE]G motif are located at the N-end and C-end of each short matrix helix, respectively, and the DCxx[RK] motif is located on each matrix helix near its N-end [8,9,12,14]. The [YWF][KR]G motif forms a featured β-turn structure before the N-end of each even-numbered helix, and these regions are crucial for cardiolipin binding [12,14,24]. These conserved sequence motifs and interactions among different structural elements have been thoroughly reviewed and investigated in previous work [8,16,22].

In addition to functioning as connectors, loops within proteins have also been reported to play important roles in allostery and conformational changes [25]. For AAC, previous experimental and computational studies have shown the potential involvement of loops in ADP/ATP transport. On the cytoplasmic side, the C2 loop contributes to substrate attraction, and the C1 loop helps to stabilize the substrate recognition site based on previous molecular dynamics (MD) simulations [11]. On the matrix side, the importance of the M1 loop fluctuations in state transition was highlighted through cross-linking experiments [26], and dramatic conformational changes in the M2 loop in ADP/ATP transport were suggested by proteolysis together with Cys labeling [27,28,29]. However, potential mechanisms of how these matrix loops are involved in ADP/ATP transport remain largely elusive.

A matrix loop of AAC connects the C-end of the odd-numbered helix and the N-end of the short matrix helix within the same domain (Figure 1A,C). At the C-ends of odd-numbered helices, triplet 37 residues (V37, A142 and M239) form a hydrophobic plug that closes the pocket near the matrix side of AAC in the c-state (Figure 1C). Here, “triplet” refers to a group of three residues at symmetric positions in the tripartite structure of AAC based on the residue number in the first domain of bovine AAC1 [8]. Before the triplet 37 residues are the featured Px[DE]xx[KR]xRxQ motifs. At the C-end of a matrix loop is the [YF]xG motif, which forms a β-turn structure before the matrix helix [24] (Figure 1C). Moreover, a previous study showed that R151 in the M2 loop forms a nice stacking structure with the capping arginine R30 of the Px[DE]xx[KR]xR motif and the middle residue R71 of the [YWF][KR]G motif [8,30], while the M3 loop intervenes between D167 of the [DE]G motif and capping arginine R139 [22]. These observations indicate that matrix loops have a close relationship to the conserved MCF motif residues near the matrix side, and we hypothesize that interacting with the conserved motif residues could be an important way for the matrix loops to participate in the function of AAC.

The unliganded AAC predominantly adopts the c-state conformation in physiological conditions [31,32]. Therefore, the sequence–structure relationship of AAC in the c-state is of fundamental significance in exploring the ADP/ATP transport mechanism [7,11,22,24,30,33,34]. In the current work, the structures and dynamics of the matrix loops in bovine AAC1 (SLC25A4) in the c-state and their relationships to the conserved sequence motifs near the matrix side were analyzed at the atomic level through MD simulations of 12.4 μs in total. Our results highlight asymmetry in the three matrix loops and reveal potential mechanisms of their participation in ADP/ATP transport.

## 2. Results

### 2.1. Both Intra-Domain and Inter-Domain Interactions Are Asymmetrically Distributed in the Three Matrix Loops of AAC in the C-State

To investigate interactions between matrix loops and conserved MCF motif residues in atomic detail, we first ran three parallel 3 μs MD simulations on c-state *apo* AAC embedded in a mixed lipid bilayer containing cardiolipins. The results show that the overall structure of AAC is stably maintained throughout the simulations, but the structural dynamics of the three matrix loops are quite different. Based on RMSF calculations, the M1 and M3 loops show conformational heterogeneity in different trajectories, while the M2 loop in all simulations consistently shows low flexibility (Figure S1). As reported in our previous work, the M2 loop is fixed in the crystal conformation (PDB ID: 1okc [12]) due to the presence of the R30:R71:R151 stacking structure [30]. 

**Figure 2:**
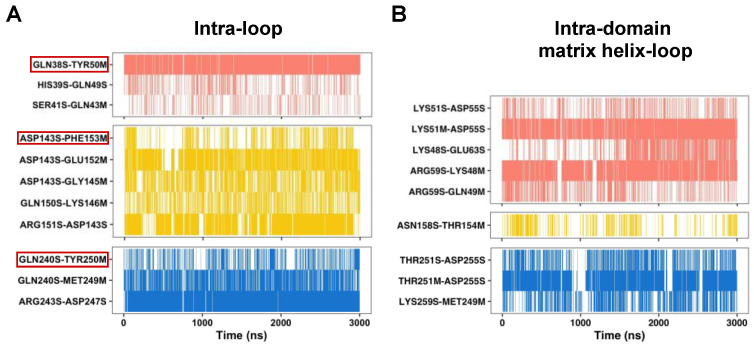
Time evolutions of the intra-domain H-bonds near the matrix side of AAC. (**A**) The intra-loop H-bonds. The H-bonds formed between triplet 38 residues (Q38, D143 and Q240) and the first residues within the [YF]xG motif (Y50, F153 and Y250) are highlighted with red boxes. (**B**) The intra-domain H-bonds formed between matrix helices and matrix loops. The H-bonds in domains 1, 2 and 3 are shown in salmon, yellow and blue, respectively. Results shown in this figure were calculated based on traj-1, and results of traj-2 and traj-3 are provided in the Supplementary Material.

Then, we screened all the interactions within each loop, the intra-domain interactions between the matrix loop and matrix helix, and the inter-domain interactions at the three domain–domain interfaces. To simplify the analysis, here, we only focus on those interactions with average occupancy higher than 25%. Our results show that all these interactions are asymmetrically distributed in the three domains. The strongest intra-loop interactions are observed within the M2 loop in all simulations (Figure 2A and Figure S2A,C), which is consistent with the fact that the conformation of the M2 loop is pretty defined. The first domain shows the strongest interactions between the matrix loop and matrix helix (Figure 2B and Figure S2B,D). At the three domain–domain interfaces, the domain 2 and 3 interface consistently exhibits strong inter-domain interactions in three parallel simulations (Figure S3).

### 2.2. Two Ends of a Matrix Loop Are Tethered through Intra-Domain Interactions between Triplet 38 Residues and Triplet 50 Residues within the [YF]xG Motif 

**Figure 3:**
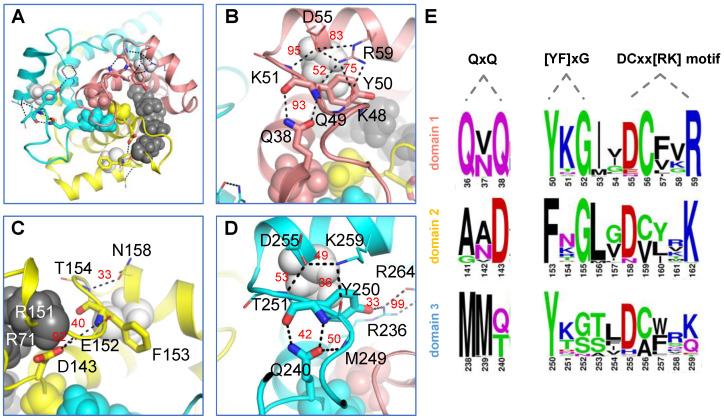
Intra-domain interactions between matrix loops and conserved MCF motifs in AAC. (**A**) Overall electrostatic interactions between matrix loops and conserved MCF motifs from the matrix side view. The conserved cysteine residues within the matrix helices, the R30:R71:R151 stacking structure and the hydrophobic plug are shown in white, black and colored spheres, respectively. Detailed interactions in domain 1, domain 2 and domain 3 are shown in (**B**–**D**), respectively. Electrostatic interactions are represented by black dashed lines, with the occupancies shown in red numbers. (**E**) Sequence logos of the QxQ motif, [YF]xG motif and DCxx[RK] motif in orthologs of AAC. The logos were generated by 43 sequences of various AAC subtypes from a total of 24 species (all sequences have been reviewed in UniProt). The residues are numbered based on bovine AAC1. In (**A**–**D**), the structures of domains 1, 2 and 3 are shown in salmon, yellow and blue, respectively.

The above analysis of the intra-loop interactions reveals consistent H-bonds between triplet 38 residues (Q38, D143 and Q240) and triplet 50 residues (Y50, F153 and Y250) within the [YF]xG motif (Figure 2A and Figure S2A,C). The triplet 38 residues follow after the hydrophobic plug formed by triplet 37 residues and become the N-end of each matrix loop. The residues within the [YF]xG motif form a β-turn structure at the C-end of each matrix loop. Therefore, the conserved H-bonds between triplet 38 residues and triplet 50 residues help tether together the two terminal ends of each matrix loop in a typical Ω loop. This is a special class of loops with short and specific distances between the two ends of the loops (Figure 3A). The Ω loops have been reported to be of significance to regulatory functions and biomolecular recognition [25]. We also observed that the strength of these H-bonds within the three domains is quite asymmetric. The M1 loop shows much stronger interactions between triplet 38 residues and triplet 50 residues than the other two matrix loops (Figure 2A and Figure 3B–D). Worthy of mention, because of the limited space caused by the R30:R71:R151 stacking structure, a smaller aspartic acid residue (D143) appears at the triplet 38 position in domain 2. D143 is strictly conserved in all AACs from different species (Figure 3E), and its interaction with R151 could be an important factor in stabilizing the R30:R71:R151 stacking structure (Figure 2A and Figure 3C). Although D143 does not bind strongly to the [YF]xG motif, it binds to the backbone amide group of E152 with a high average occupancy of 92%. 

### 2.3. Intra-Domain Interactions between the Matrix Loop and DCxx[RK] Motif in the Matrix Helix

In addition to the stabilizing effect of triplet 38 residues, the C-end of the matrix loop is also stabilized by its intra-domain interactions with the DCxx[RK] motif residues [8] at the N-terminal part of the matrix helices (Figure 3). The three cysteine residues in these motifs are usually used in cross-linking experiments to explore conformational changes during state transitions of AAC [9]. In each domain of the c-state crystal structure, the conserved cysteine within the DCxx[RK] motif forms SH–π stacking interaction with the side chain of the aromatic residue in the [YF]xG motif. This SH–π stacking structure is largely maintained in MD simulations. This stacking structure forms the hydrophobic core between the matrix helix and odd-numbered helix and may help stabilize the special structural scaffold near the matrix side of each domain. In both domains 1 and 3, D in the DCxx[RK] motif forms a H-bond with backbone amide group of triplet 51 residues, and K/R in the DCxx[RK] motif forms a H-bond with the backbone carbonyl oxygen of triplet 48 or 49 residues. Meanwhile, the two charged residues in the DCxx[RK] motif also form a salt bridge with each other. However, this electrostatic network between the DCxx[RK] motif and matrix loop in domain 1 is much stronger than that in domain 3 (Figure 2A and Figure 3B,D), and the major reason is that R (R59) appears in the DCxx[RK] motif of domain 1, while K (K259) appears in the same position of domain 3. The interactions between the C-end of the M2 loop and the N-end of the h34 helix in the domain 2 are much weaker than those in the other two domains (Figure 2A and Figure 3C). The above results show that electrostatic networks formed by the DCxx[RK] motif and matrix loop are also asymmetrically distributed in the three domains.

### 2.4. Initial Progression Direction of a Matrix Loop Is Determined by Interactions between [DE]G Motif and Capping Arginine

**Figure 4:**
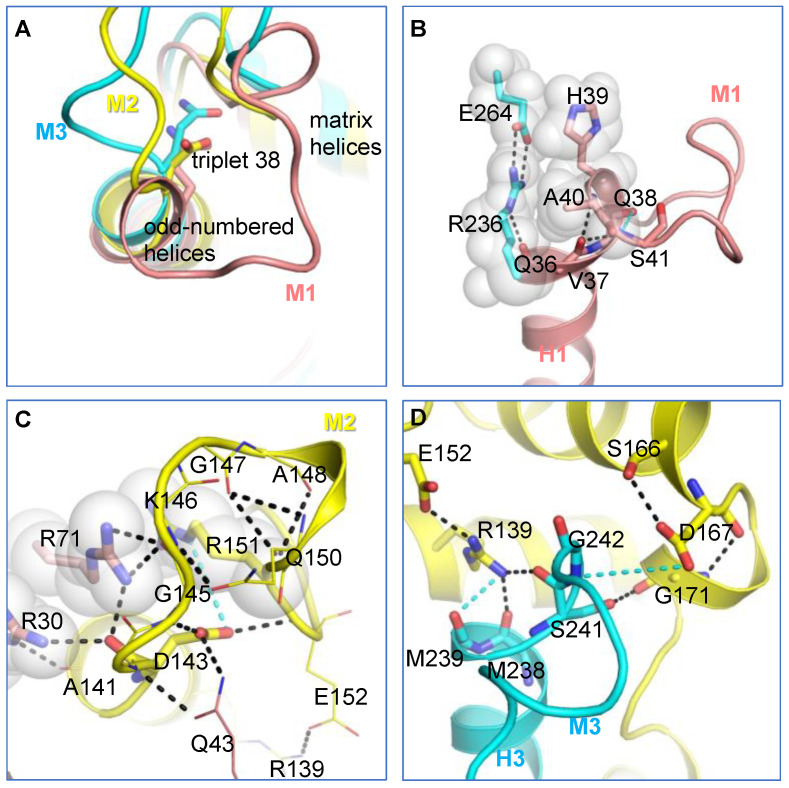
The determinants of the initial progression direction of the matrix loops of AAC. (**A**) Superimposed structures of the three domains to highlight the different progression directions of the three matrix loops. Residues of triplet 38 are presented in stick mode. Detailed interactions in domain 1, domain 2 and domain3 are shown in (**B**–**D**), respectively. Residues that cause steric hindrance (**B**) or form a stacking structure (**C**) are also shown as transparent spheres. In (**A**–**D**), the structures of domains 1, 2 and 3 are shown in salmon, yellow and blue, respectively.

Following the triplet 38 residues, the three matrix loops propagate in quite different ways (Figure 4A). For the M1 loop, the backbone carbonyl oxygen of V37 forms H-bonds with backbone amide groups of both A40 and S41 in all three parallel simulations, and meanwhile, the backbone carbonyl oxygen of Q38 forms a dynamic H-bond with the backbone amide group of S41 (Figure 4B and Table 1). Because of these H-bonds, the N-end of the M1 loop forms an extended helical structure of H1 in all our simulations on c-state bovine AAC1 (Figure S4A). The extended helical structure of H1 is different from the crystal structures of bovine AAC1 (Figure S4B) but resembles the crystal structures of yeast AAC (Figure S4C). Whether forming a helical structure or not, the progression directions of the N-end of the M1 loop are similar (Figure S4), and we speculate that the progression direction is mainly determined by the strong R236:E264 salt bridge in domain 3. This salt bridge forms a specific H-bond with the exposed backbone carbonyl oxygen of Q36 and also brings steric hindrance to residues H39 and A40 in the M1 loop (Figure 4B).

It was reported that in the presence of cardiolipin, the conformation of the M2 loop is pretty defined because of the featured R30:R71:R151 stacking structure [30]. R30 binds to the backbone carbonyl oxygens of both A141 and D143, and R71 binds to the backbone carbonyl oxygens of both D143 and G145 (Figure 4C and Table 2). These H-bonds determine the initial progression direction near the N-end of the M2 loop. R151 within the stacking structure is the key factor that determines the overall conformation of the M2 loop. The extensive electrostatic interactions within the M2 loop are shown in Figure 4C.

The M3 loop is unique in that its N-terminal side intervenes between D167 of the [DE]G motif and the capping arginine R139. Actually, D167 and R139 work like a clamp to fix the N-end of the M3 loop through an exquisite electrostatic network that includes R139, D167, S166, G171, S241 and G242 (Figure 4D). All residues involved in this network are highly conserved in AACs, suggesting that such a clamped structure could be of functional significance.

### 2.5. Impact of the E264A Mutation on the Structure of AAC

The R236:E264 salt bridge is extremely stable in MD simulations, and the two residues are strictly conserved in different AAC isoforms from different species. As shown in Figure 4B, the stable R236:E264 salt bridge is suggested to be important in shaping the progression direction of the M1 loop. Moreover, we also proposed previously that [DE]G motifs are necessary for stabilizing the capping arginine residues, which are crucial in stabilizing the C-ends of the odd-numbered helices [22].

**Figure 5:**
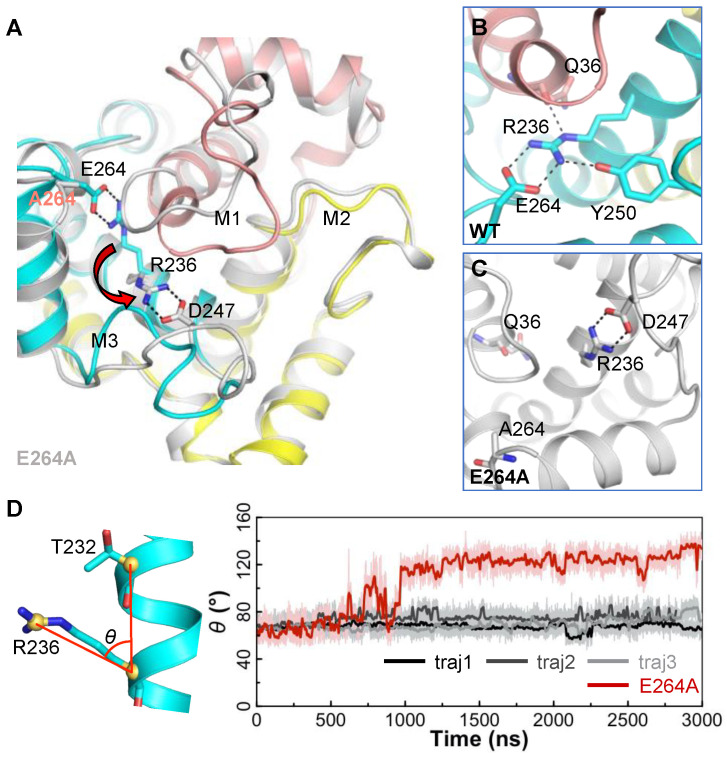
Impact of the E264A mutation on the structure of AAC. (**A**) Superimposed structures of the wild-type AAC (in color) and E264A-AAC mutant (in white) at the end of the 3 μs simulations. The flip of the R236 side chain from wild-type conformation to the E264A mutant conformation is highlighted by a red arrow. (**B**,**C**) The detailed electrostatic interactions involving R236 in the wild-type AAC and E264A-AAC mutant. These interactions are shown by black dash lines. (**D**) Time evolutions of the side-chain orientation of R236 (*θ*) during the simulations of the wild-type AAC and E264A-AAC mutant. Here, *θ* is defined as the angle between the Cζ atom of R236, the Cα atom of R236 and the Cα atom of T232 (R236Cζ- R236Cα- T232Cα). In (**A**,**B**), the structures of domains 1, 2 and 3 of wild-type AAC are shown in salmon, yellow and blue, respectively.

To test these hypotheses, we introduced the E264A mutation in AAC and ran a 3-μs MD simulation on the mutant. The results show that the mutation causes big changes in the conformations of the M1 and M3 loops (Figure 5A). In the wild-type AAC, the R236:E264 salt bridge together with Q36 and Y250 forms a stable electrostatic network (Figure 5B). In the E264A-AAC mutant, without the negatively charged residue in the [DE]G motif, although the capping arginine R236 still binds to the backbone carbonyl oxygen of Q36 at the beginning of the simulation, the interaction is much more dynamic than that in the wild-type AAC, and at 968 ns, R236 changes its side-chain orientation (Figure 5D) and starts to bind stably to D247 in the M3 loop, which leads to a big change in the conformation of the M3 loop (Figure 5A). Meanwhile, when R236 binds to D247, a big gap forms between R236 and A264 (Figure 5C). H1 extends its helical structure and fills this gap, and therefore, the conformation of the M1 loop changes drastically (Figure 5A). In contrast to the M1 and M3 loops, the M2 loop is not affected by the E264A mutation, and it maintains the stable conformation throughout the simulation because of the presence of the R30:R71:R151 stacking structure, as mentioned above. Therefore, the above results support our hypotheses: the strong R236:E264 salt bridge is a key factor in determining the orientation of the M1 loop near its N-end, and R236 itself is not enough to stabilize the C-end of H1.

### 2.6. MD Simulations of the E264D-AAC and D167E-AAC Mutants Suggest D and E in the [DE]G Motif Are Quite Different

The above results highlight the importance of the interaction between the negatively charged residue in the [DE]G motif and the capping arginine of the same domain to the progression direction of the matrix loop in the next domain. D167 and E264 belong to the [DE]G motifs in domains 2 and 3 respectively, and these two residues are strictly conserved in AACs. The strong E264:R236 salt bridge in domain 3 determines the progression direction of the M1 loop mainly through steric hindrance (Figure 4B), while D167 and R139 in domain 2 determine the progression direction of the M3 loop by binding the loop from the two sides (Figure 4D). Although Glu (E) and Asp (D) are similar in chemical properties, the side chain of E is longer than that of D by one methylene group. The strict conservation of D in position 167 and E in position 264 and their different interactions with the matrix loops lead us to speculate that D and E in the [DE]G motif are not equivalent, and the choice of D or E in different domains could possibly be related to the transport mechanism. 

**Figure 6:**
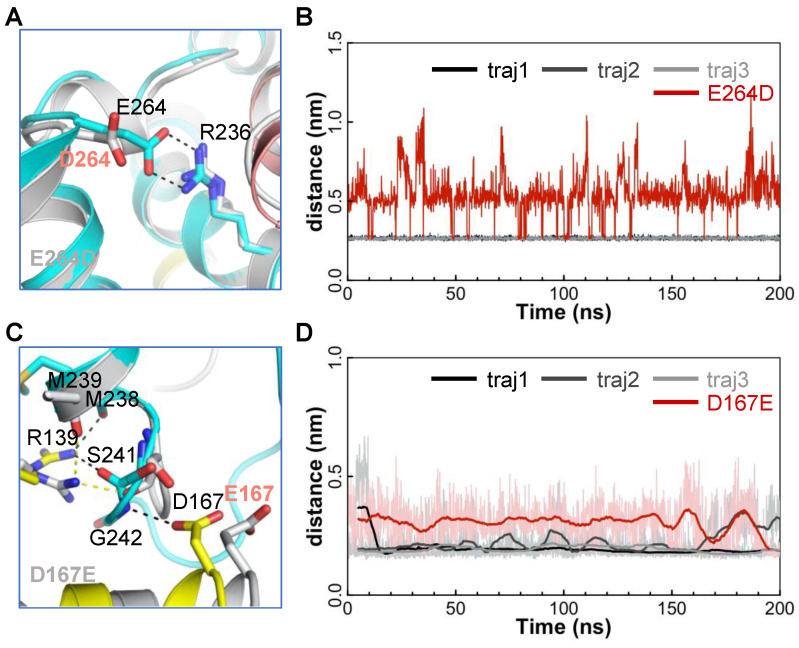
Structural changes in the simulations of E264D-AAC and D167E-AAC mutants. (**A**) Salt bridges between R236 and E/D264. (**B**) Time evolutions of minimum distances between residues 236 and 264 in the wild-type AAC (dark lines) and E264D-AAC mutant (red line). (**C**) The N-end of the M3 loop forms interactions with D/E167 and R137. (**D**) Time evolutions of minimum distances between residue 167 and the M3 loop in wild-type (dark lines) and D167E mutant AACs (red line). In (**A**,**C**), the structures of domains 1, 2 and 3 are shown in salmon, yellow and blue, respectively.

To test these hypotheses, we built two mutants: E264D-AAC and D167E-AAC. The 200 ns MD simulations of the E264D-AAC mutant show that D264 cannot bind to R236 as E264 does (Figure 6A,B). Meanwhile, the 200 ns MD simulations of the D167E mutant show that E167 does not bind to the M3 loop as D167 in the wild-type AAC does (Figure 6C,D), but R139 still binds stably to the M3 loop from the other side with a high occupancy of 74%. Therefore, our results suggest that the distance between the [DE]G motif and the capping arginine is perfect for accommodating the E:R salt bridge but a bit too long for the formation of the D:R salt bridge. AAC could take full advantage of this difference between D and E in the [DE]G motif to facilitate its transport.

**Figure 7:**
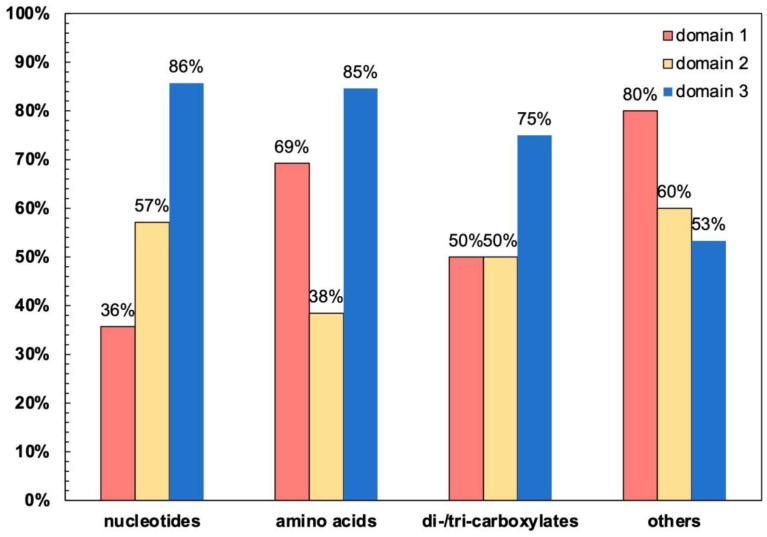
Frequencies of E in the [DE]G motifs of the three homologous domains in different groups of human MCs. Of a total of 53 human mitochondrial carriers, 46 were used for the calculation. Those carriers that are reported to localize outside the IMM (MTCH1, MTCH2, SLC25A46 and PM34) and those having an unexpected charged residue in the second position of the [DE]G motif (SLC25A47, SLC25A51 and SLC25A52) were excluded from the calculation. Please refer to Supplemental Table S1 for detailed information on the members of each group and the first residues of the [DE]G motifs in the three homologous domains of each carrier.

In the 46 tested members of human MCs, at least one domain carries E in the [DE]G motif and therefore could form a strong salt bridge with the capping Arg (Table S1). In contrast, D does not always appear in MCs. Moreover, the frequencies of E in the [DE]G motifs of different domains vary a lot among different types of MCs (Figure 7). These observations, together with the above MD simulation results, strongly indicate that different choices of E or D in [DE]G motifs are related to the function of the carrier. 

## 3. Discussion

**Figure 8:**
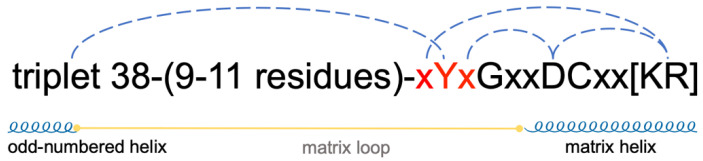
Interactions between a matrix loop and the conserved MCF motif. Electrostatic interactions are highlighted with blue dash lines. A residue is shown by a red letter if it is involved in the interactions through its backbone atoms.

In the current work, interactions between the matrix loops and conserved MCF motif residues were investigated through MD simulations of 12.4 μs in total on AAC in the c-state. Potential interactions are summarized in Figure 8. The [YF]xG motif residues at the C-end of each matrix loop forms an electrostatic network with both the N-end of the same loop (triplet 38 residues) and the DCxx[RK] motif residues at the N-terminal part of the matrix helix within the same domain. We believe that this intra-domain electrostatic network not only restricts the conformational space of the matrix loop but also helps maintain the structural scaffold between the matrix helix and odd-numbered helix near the matrix side of each domain, especially during state transitions. Of special significance, these interactions are asymmetrically distributed in three homologous domains, with the strongest network observed in domain 1 of AAC in the c-state (Figure 2 and Figure 3). Moreover, this network is also observed in domain 1 of the m-state crystal structure of AAC (Figure S5). Meanwhile, previous cross-linking experiments suggested drastic movement of domain 1 during state transitions in AAC [26,35]. Based on these results, we speculate that the strong intra-domain electrostatic network in domain 1 could be an effective way to maintain the local structural scaffold of this domain during its drastic movement. 

Another interesting finding of this work is the huge difference in the chemically similar D and E in the [DE]G motif: E in the [DE]G motif can form a perfect salt bridge with the capping arginine, but D is not long enough to form a similar salt bridge. Our results suggest that the strong R236:E264 salt bridge is a key factor in stabilizing the C-end of H1 and determining the initial orientation of the M1 loop (Figure 4B). Without the presence of the R236:E264 salt bridge in the E264A-AAC mutant, H1 exhibits an inclination to extend its helical structure (Figure 5A). Meanwhile, the R236:E264 salt bridge is also observed in the m-state crystal structure of AAC [15], which implies that this salt bridge is stably maintained during state transitions. Moreover, previous experiments suggested that domain 1 is exposed in the m-state and the M1 loop plays a gating role in closing the c-state [29]. All of these observations lead us to infer that the extremely stable R236:E264 salt bridge in domain 3 could be crucial in terminating H1 at the appropriate place and shaping the M1 loop when domain 1 moves back from the exposed conformation in the m-state to the closed conformation in the c-state. 

**Figure 9:**
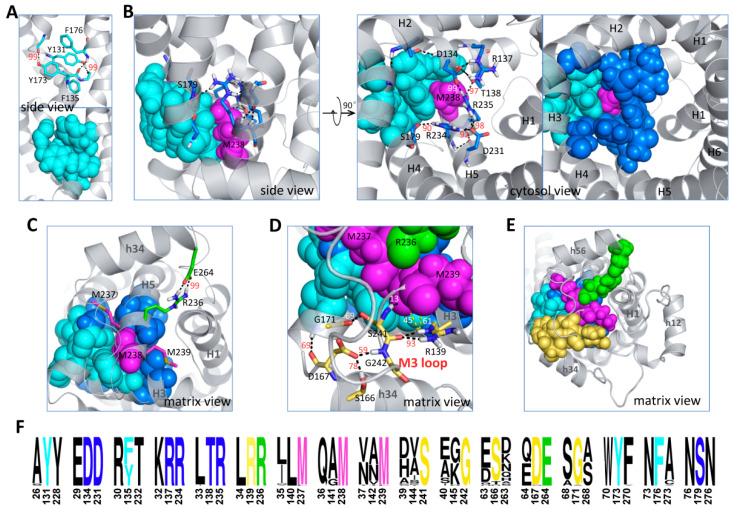
A highly asymmetric stable core exists in domains 2 and 3 of c-state *apo* AAC. (**A**) The interlocked aromatic cluster formed between the [YFW][KR]G motif residues and proline kink residues in domain 2. The side chains of C128 and Q174 are not shown for clarity purposes. (**B**) The interlocked aromatic cluster and the H3H4H5 network wrap tightly around M238. Occupancies for some bonds are shown in red or white numbers, and for complete data, please refer to our previous work [22]. (**C**) The matrix view of the positions of M237, M239 and the stable R236:E264 salt bridge. (**D**) The N-end of M3 is clamped by domain 2 through an exquisite electrostatic network. (**E**) The c-core residues are asymmetrically distributed. (**F**) The sequence logo presentation of the c-core residues within their triplets. In (**A**–**F**), the interlocked aromatic cluster in domain 2 is shown in cyan; the H3-H4-H5 network is shown in blue; MMM is shown in magenta; the R236:E264 salt bridge is shown in green; and the network between domain 2 and the N-end of the M3 loop is shown in light orange. Residues are shown first as sticks (with only polar hydrogen atoms) to highlight the interactions and then in spheres (with all hydrogen atoms) to show the space occupancy.

The choice of D (D167) in the [DE]G motif of domain 2 leads to a space between D167 and capping arginine R139 so that the N-end of the M3 loop can be inserted in between and form the clamped structure shown in Figure 4D. Such a clamped structure substantially strengthens the interaction between domain 2 and domain 3. Of special significance, our previously reported interactions between different MCF motif elements also exhibit the highest stability within domains 2 and 3 [22]. As reported, the interlocked aromatic cluster consisting of Y131, Y173, F135 and F176 (Figure 9A) makes the interaction between the Pro kink and the [YWF][KR]G turn in domain 2 much stronger than that in domain 1 or 3 [22], and this interlocked aromatic cluster is located at the domain 2–3 interface. The broad m-gate network also shows the strongest interaction between H3 and H5, and H1 only has weak interactions with H3 or H5 [22,34]. The R235:D134 salt bridge plays a central role in the asymmetric m-gate network. This salt bridge is prevented from forming in the CATR-inhibited crystal structure but is extremely stable in all simulations of c-state *apo* AAC, either with or without the presence of cardiolipins [30]. The R235:D134 salt bridge not only connects H5 and H3 strongly together but also determines the geometry of R234 and makes R234 a strong connection between H5 and H4. Therefore, R234 and R235 in the RRRMMM motif are crucial in attaching helices H3, H4 and H5 together near the m-gate level, and this H3-H4-H5 network, involving D134, R137, S138, S179, D231, R234 and R235, forms the most stable part of the broad m-gate network (Figure 9B). Of special interest, the above-mentioned interlocked aromatic cluster and the H3-H4-H5 network form a circle and wrap tightly around the long hydrophobic side chain of M238 (Figure 9B), the middle methionine of the RRRMMM motif. Outside the pocket, the other two methionines of the RRRMMM motif are perpendicular to the M238 side chain (Figure 9C). Therefore, the three methionines form a T-shaped structure, and their hydrophobic side chains may introduce considerable stability via van der Waals interactions with neighboring residues. M237 is located at the domain 2–3 interface, while M239 together with V37 and A142 forms the hydrophobic plug that seals the odd-numbered helices near the matrix side. Adjacent is the extremely stable R236:E264 salt bridge, the strongest interaction formed between the capping arginines [22] and the [DE]G motifs in the three domains (Figure 9C). Moreover, as mentioned above, the capping arginine R139 and D167 in the [DE]G motif work like a clamp to fix the N-end of the M3 loop through an exquisite electrostatic network involving R139, D167, S166, G171, S241 and G242 (Figure 9D). S241 and G242 of the M3 loop follow shortly after the RRRMMM motif; therefore, such a clamped structure not only improves the interaction between domain 2 and domain 3 but also helps maintain the conformations of MMM. Figure 9E clearly shows that all the above stable structural elements cluster together to form a stable core within domains 2 and 3 of AAC in the c-state. This stable core holds domains 2 and 3 tightly together near the matrix side, and obviously, the RRRMMM motif is the central part of this stable core. Of special interest, each residue of this stable core is extremely conserved and highly asymmetric within the triplets [8] (Figure 9F), which strongly implies that this stable core and its asymmetric distribution are of significance to the transport mechanism of the carrier. 

The presence of the stable core within domains 2 and 3 suggests that although the structural scaffold of AAC in the c-state is pseudosymmetric, its structural dynamics is highly asymmetric. Moreover, previous simulations suggest that the specifically bound cardiolipins also strengthen the interactions between domain 2 and domain 3: the cardiolipin bound at the domain 2-3 interface predominantly adopts the inter-domain binding mode, while cardiolipins at the other two interfaces have much more intra-domain populations [24]. The asymmetry of the intrinsic dynamic properties of AAC in the c-state and the cardiolipin binding behavior support the asymmetric crystal structure of m-state AAC, in which domains 2 and 3 still attach together, while domain 1 is isolated from the other two domains on the matrix side [15]. The displacement of domain 1 in the m-state structure is also supported by early cross-linking experiments, which reported that domain 1 is exposed in the m-state and undergoes drastic movement during state transitions [26,35]. The above observations indicate that domain 1 and domains 2 and 3 of AAC work as two structural units during transport. Considering the specific choices of the chemically similar D or E in the [DE]G motif in different domains, we propose that AAC may take full advantage of the asymmetry between the three homologous repeat domains to improve its transport efficiency. 

## 4. Materials and Methods

### 4.1. Preparation of the Wild-Type AAC Systems and Equilibration Steps of MD Simulation

The initial coordinates of the carrier were obtained from the AAC–CATR complex structure of Bos taurus (PDB entry: 1okc) [12] with coordinates of the missing residues of AAC built with iTasser [36]. The inhibitor CATR was removed from the system, and the crystal water molecules were kept. The two terminal residues of the carrier were set to the charged state. The CHARMM-GUI Membrane Builder [37,38,39,40] was used to build a 12.5 nm × 12.5 nm mixed lipid bilayer composed of POPC (1-palmitoyl-2-oleoyl-sn-gly cero-3-phosphocholine), POPE (1-palmitoyl-2-oleoyl-sn-glycero-3-phosphatidylethanolamine) and cardiolipin (CL,1′,3′-Bis [1,2-dilinoleoyl-sn-glycero-3-phospho]-glycerol) in a molar ratio of 5:4:1. Each type of lipid was equally distributed in the two leaflets of the bilayer. As the three specifically bound CLs are incomplete in the crystal structure, they were replaced with the three intact CL molecules. Here, the structure of the intact CL molecule was picked from the above-mixed lipid bilayer and fitted manually to the incomplete CL in the crystal structure within PyMOL [41]. A hole was then made in the mixed lipid bilayer, and AAC with three bound CLs was embedded manually into the mixed bilayer through PyMOL. The system was solvated with TIP3P water molecules [42]. After net neutralization of the system with Na^+^, additional NaCl was added to reach a concentration of 50 mmol/L. This led to an 82971-atom system consisting of one wild-type AAC protein, 110 POPCs, 88 POPEs, 22 CLs, 15740 waters, 53 Na^+^ and 28 Cl^−^.

After the wild-type system was set up, energy minimizations and dynamic equilibrations were carried out with the GROMACS 4.5.5 package [43] with the CMAP-modified [44] CHARMM36 force field [45]. Periodic boundary conditions were applied for simulation systems. Four rounds of energy minimizations were first performed to remove unfavorable contacts and prevent the AAC–lipid system from collapsing in the subsequent dynamic equilibrations. Positional restraints were sequentially applied on all of the heavy atoms, main-chain atoms and Cα atoms of AAC using a force constant of 1000 kcal/mol/Å^2^. In the final minimization step, no positional restraints were applied. Following the energy minimizations, four rounds of equilibration steps were carried out. In the first step, the system was heated from 50 K to 310 K in the NBV ensemble with positional restraints applied on heavy atoms of the protein; the time step was set to 1 fs, and the simulation time was set to 10 ns. The second equilibration step was as long as 160 ns to fully equilibrate the lipid bilayer and the solvent, with positional restraints applied on all main-chain atoms of the protein, and the time step was set to 2 fs. In the subsequent two steps, the system was equilibrated with positional restraints on all Cα atoms of AAC and with no positional restraints, respectively, with a time step of 2 fs and simulation time of 10 ns for each step.

### 4.2. Preparation of Mutant AAC Systems and Equilibrations

The initial coordinates of the three AAC mutant systems (E264A-AAC, E264D-AAC and D167E-AAC) were built based on the above wild-type AAC system after its second equilibration step, in which the lipid bilayer and the solvent were fully equilibrated. For the carrier, the mutations were introduced through PyMOL [46], with the coordinates of lipids and solvent kept from the equilibrated wild-type AAC system. In the E264A-AAC system, the mutation led to one extra positive charge, and we hence removed one sodium ion in the bulk solvent to balance the charge. In the D167E-AAC system, to minimize the disturbance from charged residues in the M3 loop to E167, we also mutated all these residues in the M3 loop to alanines (R243A, K244A and D247A). Then, the mutant systems underwent two rounds of quick equilibration steps: each system was first heated from 50 K to 310 K with positional restraints applied on heavy atoms of the protein with a time step of 1 fs, and the simulation time was 100 ps. Then a 100 ps equilibration step was carried out with the same restraint strategy with a time step of 2 fs.

### 4.3. Production of MD Simulation and Trajectory Analyses

After equilibration, three independent 3 μs production simulations were run in parallel for the wild-type AAC system. One 3 μs production simulation was carried out for the E264A-AAC system, and one 200 ns simulation was carried out for each of the E264D-AAC and D167E-AAC mutant systems. The pressure of each system was maintained at 1 bar with the Berendsen method [47], τp was set to 1.0 ps, and compressibility was set to 4.5 × 10^−5^ bar^−1^. The temperature was maintained at 310 K with the v-rescale method [48], and the coupling time was set to 0.1 ps. The SETTLE [49] and LINCS constraints [50] were applied on hydrogen-involved covalent bonds in water molecules and in other molecules, respectively. Electrostatic interactions were calculated with the Particle-Mesh Ewald (PME) algorithm [51]. The coordinates of each system were saved every 10 ps.

Trajectories were analyzed mostly with programs provided in the GROMACS 4.5.5 package. Occupancies of salt bridges and H-bonds were calculated based on the last 2 μs of each trajectory, with a distance cut-off set to 0.33 nm. Trajectories were displayed with VMD [52], and structure representations were prepared with PyMOL [41]. The sequence logos were generated with WebLogo [53]. The same trajectories of the wild-type AAC have already been used to describe CL binding modes [24] and assess the effect of CL binding on the structural dynamics of AAC in the c-state [30].

## Figures and Tables

**Table 1:** Occupancies (in percentage) of crucial interactions associated with the initial progression directions of the matrix loops.

	Traj-1	Traj-2	Traj-3	Average
V37(O):A40(NH)	76	58	66	67
V37(O):S41(NH)	8	14	12	11
Q38(O):S41(NH)	21	30	13	12
R30:A141(O)	98	100	99	99
R30:D143(O)	18	16	40	25
R71:D143(O)	78	74	36	63
R71:G145(O)	99	99	78	92

**Table 2:** Summary of simulations in the current work.

System	Mutations	Simulation Time
Wild-type AAC	/	3 × 3 μs
E264A-AAC	E264A	3 μs
E264D-AAC	E264D	200 ns
D167E-AAC	D167E	200 ns

## Data Availability

Data are available upon reasonable request to the corresponding author.

## Supplementary Materials

The supporting information can be downloaded at: https://www.mdpi.com/article/10.3390/ijms231810877/s1.
