# Supplementary material for: Function-Related Asymmetry of the Interactions between Matrix Loops and Conserved Sequence Motifs in the Mitochondrial ADP/ATP Carrier
Source: Int J Mol Sci. 2022 Sep 17;23(18):10877. doi: 10.3390/ijms231810877 (PMC9502086; doi:10.3390/ijms231810877)
Supplement: Supplementary file 1 [file ijms-23-10877-s001.zip › ijms-1855745-supplementary.pdf]

Supplementary information

Function-related asymmetry of the interactions between matrix loops and conserved sequence motifs in the mitochondrial ADP/ATP carrier

Qiuzi Yi[1,2,#], Shihao Yao[1,2,#], Boyuan Ma[1,2], Xiaohui Cang[1,2,3*]

[1]Division of Medical Genetics and Genomics, The Children's Hospital, Zhejiang University School of Medicine, Hangzhou, Zhejiang 310052, China

[2]Institute of Genetics, and Department of Genetics, Zhejiang University School of Medicine, Hangzhou, Zhejiang 310058, China

[3]Zhejiang Provincial Key Lab of Genetic and Developmental Disorder, Hangzhou, Zhejiang 310058, China.

*Corresponding author:

Xiaohui Cang
Biological Experimental Building 510-3
Institute of Genetics and Department of Genetics,
Zhejiang University School of Medicine,
Hangzhou, Zhejiang 310058, China
E-mail: xhcang@zju.edu.cn

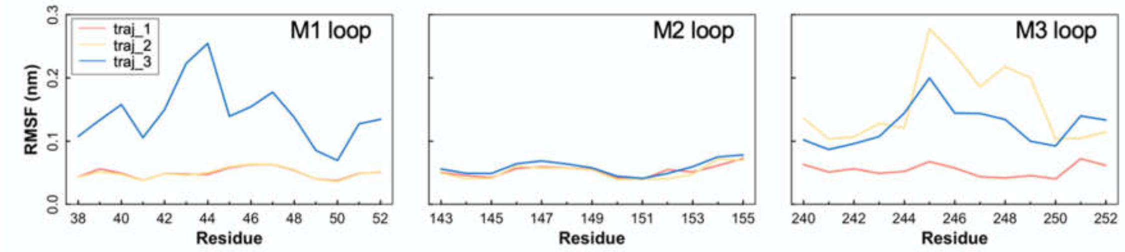

Figure S1. Root mean square fluctuation (RMSF) values of backbone atoms of the three matrix loops of AAC in the m-state.

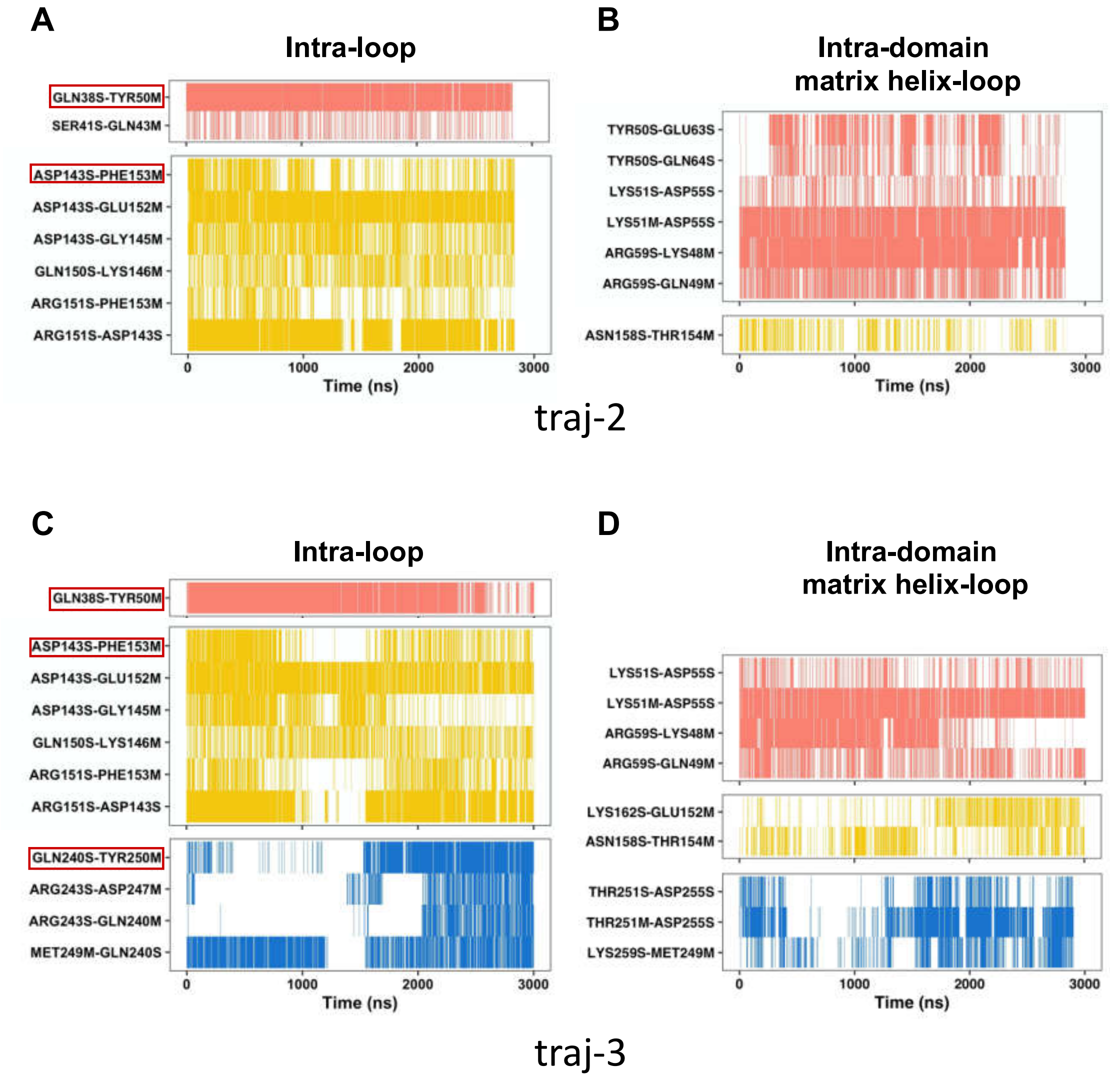

Figure S2. Time evolutions of the intra-domain H-bonds near the matrix side of AAC in traj-2 and traj-3. (A) (C) The intra-loop H-bonds. The H-bonds formed between triplet 38 and [YF]xG motif are highlighted with red rectangles. Results are not shown for the M3 loop in traj-2 as there are no strong intra-loop H-bonds. (B) (D) The intra-domain H-bonds formed between matrix helices and matrix loops. Results in domains 1, 2 and 3 are shown in salmon, yellow and blue respectively.

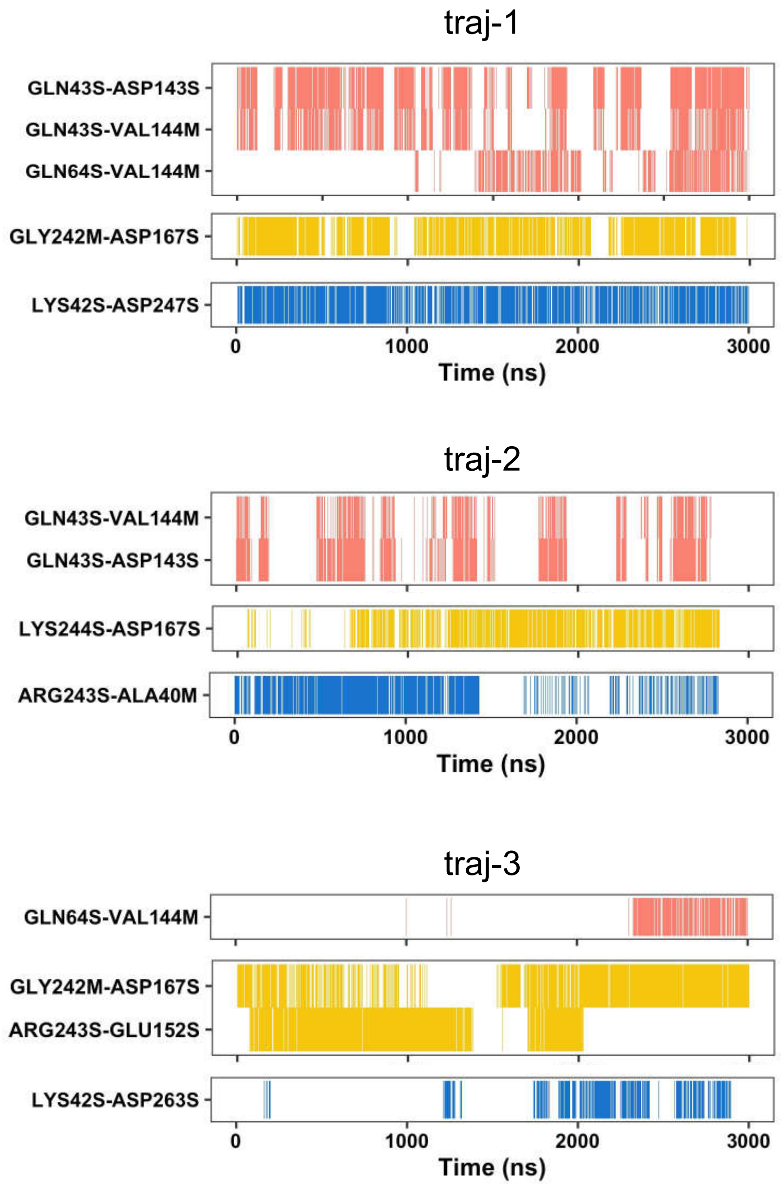

Figure S3. Time evolution of the inter-domain interactions at the three domain-domain interfaces. Domain1-2, domain 2-3 and domain1-3 interfaces are shown in red, yellow and blue, respectively.

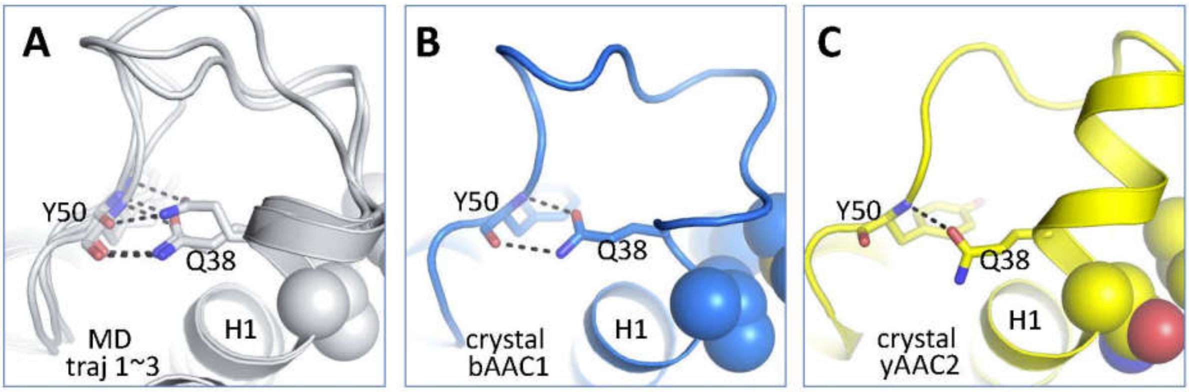

Figure S4. Conformations of the N-end of the M1 loop in three parallel MD simulations on bovine AAC1 c-state **(A)**, in the crystal structure of bovine AAC1 **(B)**, and in the crystal structure of yeast AAC2 **(C)**. Residues of the triplet 37 hydrophobic plug are shown in spheres. Q38 and Y50 are shown in sticks, and H-bonds between the two residues are shown in black dash lines.

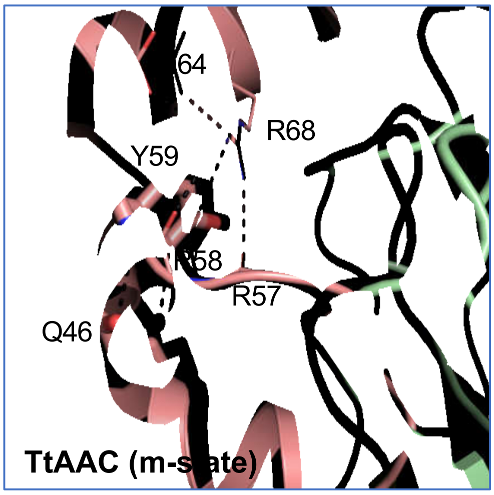

Figure S5. Intra-domain interactions between matrix loop and conserved MCF motif in domain 1 of *Thermothelomyces thermophila* AAC in the m-state.

Table S1. The first residues of the [DE]G motifs in three homologous domains of 53 human mitochondrial carriers classified in different groups.

	domain 1	domain 2	domain 3
nucleotides			
AAC1, SLC25A4	Q	D	E
AAC2, SLC25A5	Q	D	E
AAC3, SLC25A6	Q	D	E
AAC4, SLC25A31	Q	D	E
SCaMC1, SLC25A24	G	E	E
SCaMC2, SLC25A25	G	E	E
SCaMC3, SLC25A23	G	E	E
SLC25A41	G	E	Q
MFTC, SLC25A32	D	E	E
TPC, SLC25A19	E	E	E
GDC, SLC25A16	E	G	H
SLC25A33	E	E	E
SLC25A36	E	D	E
SLC25A42	E	E	E
amino acids			
ORC2, SLC25A2	V	D	E
ORC1, SLC25A15	V	D	E
GC2, SLC25A18	E	Q	E
GC1, SLC25A22	E	R	E
CACT, SLC25A20	E	F	E
CACL, SLC25A29	E	E	E
AGC1, SLC25A12	E	L	E
AGC2, SLC25A13	E	L	E
SAMC, SLC25A26	G	E	Q
SLC25A38	E	E	Y
SLC25A44	D	D	E
SLC25A45	E	E	E
SLC25A48	E	E	E
di-/tri-carboxylates			
DIC, SLC25A10	D	E	L
ODC, SLC25A21	E	W	E
OGC, SLC25A11	E	E	E
CTP, SLC25A1	H	Q	E
others			
UCP1, SLC25A7	E	E	E
UCP2, SLC25A8	E	E	E
UCP3, SLC25A9	E	E	E
UCP4, SLC25A27	E	G	E
UCP5, SLC25A14	E	E	E
MFRN1, SLC25A37	E	E	G
MFRN2, SLC25A28	E	E	N
KMCP1, SLC25A30	E	E	E
PiC, SLC25A3	D	E	L
SLC25A34	D	Q	E
SLC25A35	D	H	E
SLC25A39	E	G	S
SLC25A40	E	D	N
SLC25A43	E	E	Q
SLC25A53	E	Y	R
